# Prevalence and Clinical Correlates of Hypomagnesemia in Ischemic Stroke Patients: A Single-Center Study From a Tertiary Care Center in Pakistan

**DOI:** 10.7759/cureus.84742

**Published:** 2025-05-24

**Authors:** Saad Riaz, Murad Ali, Manzoor Hussain, Zahid Hassan, Hamidullah NA, Zia Ur-Rehman, Sajjad Ali, Izhar Ahmed, Shahid Shahzad

**Affiliations:** 1 Internal Medicine, Mardan Medical Complex, Mardan, PAK; 2 Pediatric Cardiology, Maqsood Medical Complex (MMC) General Hospital, Peshawar, PAK; 3 Pulmonology, Mardan Medical Complex, Mardan, PAK; 4 Urology, District Headquarter Hospital, Nowshehra, PAK

**Keywords:** hypertension, hypomagnesemia, ischemic stroke, pakistan, smoking

## Abstract

Background: Hypomagnesemia has been linked to stroke development through its effects on blood vessel function and nerve cell excitability, but data from South Asia are limited. The aim of this study was to determine the prevalence of hypomagnesemia in patients with acute ischemic stroke and its associated risk factors at a tertiary care hospital in Pakistan.

Methods: The study employed a cross-sectional design, conducted from November 2020 to June 2021, and enrolled 137 consecutive stroke patients. Hypomagnesemia was defined as serum magnesium levels less than 1.82 mg/dL. SPSS software v24 (IBM Corp., Armonk, NY) was used to analyze the association between hypomagnesemia and various demographic and clinical factors.

Results: Hypomagnesemia was found to be present in 48.9% of ischemic stroke patients. The most common associated comorbidities were hypertension and type 2 diabetes mellitus. The study discovered a strong correlation between hypertension and stroke, and 61.1% of the hypertensive patients with stroke had hypomagnesemia. Hypomagnesemia was found in 18.5% of smokers compared to 56.4% of non-smokers, indicating an inverse connection with smoking. The prevalence of hypomagnesemia was similar in males and females and across age groups.

Conclusion: Hypomagnesemia was present in nearly half of the patients with acute ischemic stroke, and hypertension was a strong predictor. Further study is warranted for the lower prevalence of hypomagnesemia among smokers. Secondary strokes may be prevented in high-risk individuals by routine magnesium screening and supplementation.

## Introduction

Stroke is one of the most devastating neurological emergencies worldwide, ranking as the second highest cause of death and a major contributor to long-term disability. Globally, around 89 million people have had a stroke, and 7.08 million die from it each year [1]. The burden of stroke is particularly high in low-middle-income countries, where limited healthcare infrastructure worsens outcomes [2]. In Pakistan, the annual incidence of stroke is estimated at 250 per 100,000 people [3], and stroke is the second leading cause of death with a mortality rate of about 9.3% [2]. Despite advances in acute stroke care, many survivors still experience persistent neurological problems, highlighting the need for new treatments to reduce secondary injury and improve recovery [4].

A growing body of research indicates that electrolyte disturbances, particularly low serum magnesium levels, maybe a major factor in the pathogenesis and prognosis of stroke [5]. Magnesium acts as a natural calcium antagonist, regulating vascular tone, neuronal excitability, and cellular energy consumption, all important processes involved in the ischemic cascade [6]. Multiple international studies have reported an inverse association between serum magnesium concentrations and stroke risk. Meta-analyses show that for every 0.1 mmol/L increase in serum magnesium, the incidence of stroke decreases by 13% [7]. However, these findings have primarily originated from Western populations, with little information available from South Asia, where genetics and dietary habits may influence magnesium homeostasis.

Existing regional research from Pakistan is limited. A single-center study in Lahore found hypomagnesemia in 35.5% of acute ischemic stroke patients, with a higher prevalence among those with hypertension [8]. On the other hand, research from Bangladesh reported even greater magnesium deficiency in stroke patients, exacerbating the situation further [9]. These geographic differences highlight the need for population-specific data because of variations in dietary magnesium intake, concomitant disease load, and healthcare access. Furthermore, no studies have thoroughly examined how hypomagnesemia interacts with known stroke risk factors, such as diabetes, hypertension, or smoking, in the Pakistani population.

The purpose of this study was to fill in the gaps in the existing literature by investigating the frequency of hypomagnesemia among a Pakistani cohort of stroke patients and analyzing its associations with important demographic and clinical factors. By elucidating these relationships, our findings could inform the development of targeted magnesium supplementation protocols, a low-cost, scalable intervention with the potential to enhance secondary stroke prevention efforts in resource-limited settings [10]. Given magnesium's pleiotropic neuroprotective properties and excellent safety profile, such strategies may provide a workable way to mitigate stroke-related morbidity in high-burden areas [11].

## Materials and methods

Study design and ethical framework

This was a cross-sectional study conducted at a tertiary care hospital to assess the prevalence of hypomagnesemia and its clinical correlates in patients with acute ischemic strokes. Before the commencement of the study, ethical approval was obtained from the Institutional Review Board of Bacha Khan Medical College, and the study adhered to the ethical standards outlined in the Declaration of Helsinki. Written informed consent was obtained from all participants or their legal guardians before inclusion in the study.

Study site and duration

The study was conducted on the inpatients of Medical A Ward of Mardan Medical Complex, a tertiary healthcare facility located in the Mardan district of Khyber Pakhtunkhwa, Pakistan, between November 17, 2020, and June 29, 2021. The Mardan Medical Complex serves as a major referral center for a catchment area comprising six surrounding districts of Khyber Pakhtunkhwa, receiving patients both from primary and secondary healthcare facilities across the region.

Participant selection criteria

Adult patients having clinically and radiologically confirmed ischemic stroke and presenting within 72 hours of symptom onset were included in the study. All patients having transient ischemic attacks or hemorrhagic stroke, who had received magnesium supplementation within the preceding three months, had secondary neurological deficits, were pregnant or lactating, or had end-stage renal disease were excluded from the study.

Sample size determination

The sample size was calculated using the WHO Sample Size Calculator, with an anticipated hypomagnesemia frequency of 35.5%, a 95% confidence level, and an 8% margin of error. The minimum required sample size was determined to be 137 participants. Consecutive sampling was used to enroll eligible patients.

Data collection methodology

The data collection of the study involved several key components. Clinical assessment began with the suspicion of stroke diagnosis through meticulous medical history and clinical examination, and the diagnosis of ischemic stroke was confirmed through a CT scan of the brain. Additionally, researchers documented participants' comorbidities. For the laboratory analysis, venous blood samples were collected under aseptic conditions, and serum magnesium levels were measured using a colorimetric assay. Hypomagnesemia was defined as a serum Mg²⁺ concentration of less than 1.82 mg/dL based on the existing literature [12]. Finally, the data recording process utilized standardized case report forms and included double-data entry verification to ensure accuracy.

Statistical analysis

The collected data were analyzed using SPSS Statistics software v24 (IBM Corp., Armonk, NY). Continuous variables were presented in the form of mean ± standard deviation, and categorical variables as frequencies and percentages. The distribution of the data was assessed using histograms, which confirmed approximate normality. Therefore, between-group comparisons for continuous variables were conducted using independent samples t-tests. Categorical variables were compared using the chi-square test. The threshold for statistical significance was set at p<0.05. Non-parametric tests were not required, as no substantial deviations from normality were observed in the data.

## Results

The study included 137 patients having acute ischemic stroke with a mean age of 56.0±14.5 years, comprising 46% males (n=63) and 54% females (n=74). Hypertension was the most prevalent comorbidity (69.3%), followed by diabetes (38.0%), ischemic heart disease (28.5%), and smoking (19.7%). Hypomagnesemia (serum Mg²⁺ <1.82 mg/dL) was observed in 48.9% (n=67) of participants, with an overall mean serum magnesium level of 1.93±0.73 mg/dL.

Gender-based analysis revealed a significant difference in smoking prevalence (39.7% males vs. 2.7% females, p<0.001), while no significant differences were noted in age, hypertension, diabetes, or ischemic heart disease between sexes (p>0.05) (Table 1).

**Table 1:** Demographics and clinical profile of stroke patients (n=137) *Data presented as n (%) or mean±standard deviation. Key: the Chi-square test was used for categorical variables, and the independent samples t-test was used for continuous variables. A p-value <0.05 was considered significant; a p-value <0.001 was considered highly significant.

Characteristic	Total cohort	Male (n=63)	Female (n=74)	p-value
Age (years), mean±SD	56.0±14.5	54.2±15.1	57.6±13.8	0.182
Hypertension, n (%)	95 (69.3%)	40 (63.5%)	55 (74.3%)	0.178
Diabetes, n (%)	52 (38.0%)	22 (34.9%)	30 (40.5%)	0.512
Ischemic heart disease, n (%)	39 (28.5%)	20 (31.7%)	19 (25.7%)	0.443
Smoking, n (%)*	27 (19.7%)	25 (39.7%)	2 (2.7%)	<0.001
Serum Mg (mg/dL), mean±SD	1.93±0.73	1.91±0.75	1.95±0.71	0.742

Hypomagnesemia stratified by risk factors

Hypomagnesemia was significantly associated with hypertension (61.1% vs. 21.4%, p<0.001) and non-smoking status (56.4% vs. 18.5% in smokers, p<0.001). No significant associations were found with age, gender, or diabetes (p>0.05) (Table 2).

**Table 2:** Prevalence of hypomagnesemia stratified by risk factors Hypomagnesemia+ = serum Mg <1.82 mg/dL. Key: Comparative analysis between the groups was performed using the Chi-square test for categorical variables. A p-value <0.05 was considered significant; a p-value <0.001 was considered highly significant.

Variable	Category	Hypomagnesemia+ (n=67)	Hypomagnesemia– (n=70)	p-value
Age group	30-55 years	33 (50.0%)	33 (50.0%)	0.805
	56-80 years	34 (47.9%)	37 (52.1%)	
Gender	Male	30 (47.6%)	33 (52.4%)	0.781
	Female	37 (50.0%)	37 (50.0%)	
Hypertension*	Present	58 (61.1%)	37 (38.9%)	<0.001
	Absent	9 (21.4%)	33 (78.6%)	
Diabetes	Present	21 (40.4%)	31 (59.6%)	0.119
	Absent	46 (54.1%)	39 (45.9%)	
Smoking status*	Current smoker	5 (18.5%)	22 (81.5%)	<0.001
	Non-smoker	62 (56.4%)	48 (43.6%)	

## Discussion

This cross-sectional study evaluated the frequency of hypomagnesemia in acute ischemic stroke patients and its associated risk factors in 137 patients at a tertiary care hospital in Pakistan. The key findings from this study revealed that nearly half (48.9%) of the stroke patients had hypomagnesemia, with significant associations observed for hypertension (61.1%, p<0.001) and smoking (18.5%, p<0.001). However, no significant correlations were found with age, gender, diabetes, or ischemic heart disease. Prior investigations have established a compelling link between reduced serum magnesium levels and adverse outcomes following ischemic stroke [13].

The observed 48.9% prevalence of hypomagnesemia aligns with prior studies in similar populations. For instance, Khanum et al. (2024) reported 35% hypomagnesemia in Lahore, Pakistan [8], while studies from Bangladesh documented rates as high as 72% [9]. The higher prevalence may reflect regional dietary differences, variations in cardiovascular risk profiles, or genetics.

The strong association between hypomagnesemia and hypertension (61.1%) supports existing mechanistic evidence that magnesium deficiency contributes to vascular endothelial dysfunction, increased arterial stiffness, and elevated blood pressure [14]. Our findings reinforce the hypothesis that magnesium’s vasodilatory and anti-inflammatory effects may play a protective role in stroke prevention [15].

Interestingly, smokers exhibited significantly lower hypomagnesemia rates (18.5%) compared to non-smokers (56.4%). This contrasts with some Western studies but may reflect confounding factors such as younger age, lower hypertension prevalence, or differences in smoking composition (e.g., cigarette vs. other tobacco use). Further research is needed to explore this unexpected inverse relationship.

Despite females having numerically higher hypertension (74.3% vs. 63.5%) and diabetes (40.5% vs. 34.9%), hypomagnesemia prevalence was nearly identical between genders (50.0% female vs. 47.6% male, p=0.781). This suggests that physiological magnesium regulation may differ by sex, potentially due to hormonal influences on renal excretion [16,17].

Clinical implications

Screening and Assessment

The high prevalence of hypomagnesemia observed in this study suggests that routine evaluation of serum magnesium levels should be considered for stroke patients, particularly those with concurrent hypertension. However, this could be challenging in resource-limited countries like Pakistan due to cost-effectiveness and logistical challenges.

Targeted Preventive Strategies

Hypertensive individuals may benefit from dietary or therapeutic magnesium supplementation as a potential approach to mitigate stroke risk, given the observed associations.

Examining the Smoking Paradox

The lower prevalence of hypomagnesemia among smokers in this cohort warrants further investigation to determine the underlying factors, whether related to survivorship bias or unique metabolic interactions.

Study limitations

This study had several limitations. First, the single-center design may limit the generalizability of the findings to broader populations. Second, the cross-sectional nature of the study limits the ability to establish causal relationships between serum magnesium levels and stroke severity or outcomes. Third, the consecutive sampling may have introduced selection bias, as it does not ensure a truly random or representative sample. In addition, measurement bias can not be entirely ruled out in the assessment of serum magnesium levels due to potential pre-analytical and analytical variations. Furthermore, important confounding variables, such as the use of medications (e.g., diuretics or proton pump inhibitors) that may affect magnesium levels, were not controlled for, which could influence the study outcomes. Finally, the small number of female smokers in the cohort limits the statistical power and generalizability of findings related to this subgroup, and the results should be interpreted with caution.

Future directions

Future research should focus on conducting large-scale, randomized controlled trials to assess the efficacy of magnesium supplementation in reducing the risk of secondary stroke and improving functional recovery in stroke patients. Furthermore, exploring genetic predispositions to hypomagnesemia may offer insight into population-specific risks. Investigations into dietary magnesium intake and its relationship with stroke incidence and outcomes could also help clarify the role of nutritional interventions in stroke prevention strategies.

## Conclusions

Hypomagnesemia is highly prevalent among Pakistani stroke patients, particularly in those with hypertension. The inverse association observed with smoking warrants further investigation to clarify this relationship. These findings underscore the potential role of magnesium in stroke pathophysiology and support targeted interventions for high-risk populations in resource-limited settings like Pakistan. However, structured public health initiatives and further validation through multicenter studies are essential before large-scale implementation.
